# Supplementary material for: SARS-CoV-2 reliably detected in frozen saliva samples stored up to one year
Source: PLoS One. 2022 Aug 11;17(8):e0272971. doi: 10.1371/journal.pone.0272971 (PMC9371282; doi:10.1371/journal.pone.0272971)
Supplement: S1 File — (PDF) [file pone.0272971.s001.pdf]

ID	Original test month	Original antigen concentration (pg/mL)	Original N1 CT	Retest month	Retest antigen concentration (pg/mL)	Retest N1 CT
31	December-20	791.2	24.8	November-21	N/A	24.2
42	December-20	16.5	33.2	November-21	6.3	31.9
33	December-20	53.2	29.6	November-21	118.7	28.8
77	December-20	10.9	35.3	November-21	5.1	28.8
80	December-20	1.8	38.8	November-21	0.1	Undetermined
3	December-20	15.6	29.0	November-21	81.1	33.7
37	December-20	1.5	34.6	November-21	2.2	33.5
45	December-20	113.9	26.0	November-21	252.2	24.5
62	December-20	5.8	35.6	November-21	7.4	33.2
76	December-20	N/A	20.8	November-21	6752.0	16.7
55	January-22	4.5	32.1	November-21	10.4	30.0
79	January-21	9965.2	29.6	November-21	1814.6	22.3
49	January-21	18.8	31.8	November-21	19.5	30.0
66	January-21	435.9	27.3	November-21	644.5	24.7
74	January-21	1045.9	28.3	November-21	702.0	24.6
4	January-21	N/A	21.8	November-21	5149.8	25.8
1	January-21	208.4	27.8	November-21	123.2	26.3
18	January-21	409.1	26.5	November-21	573.1	26.8
14	February-21	712.2	26.1	November-21	409.2	26.4
6	February-21	995.6	25.2	November-21	301.2	27.4
67	February-21	200.1	26.9	November-21	262.7	24.2
47	February-21	111.1	27.6	November-21	115.6	26.0
11	February-21	N/A	15.5	November-21	58670.0	16.2
23	February-21	73.3	27.4	November-21	97.6	27.3
81	February-21	17.3	38.7	November-21	0.1	Undetermined
36	February-21	15.3	31.5	November-21	38.9	30.5
78	February-21	69.0	27.7	November-21	135.9	21.1
82	February-21	3.9	32.7	November-21	below calibration range	Undetermined
12	March-21	130.6	25.8	November-21	16.9	26.3
26	March-21	13.2	29.1	November-21	N/A	28.8
52	March-21	N/A	21.3	November-21	3498.0	19.4
53	March-21	6.0	30.8	November-21	1.5	28.9
72	March-21	48.3	29.7	November-21	8.4	26.4
83	March-21	3.2	37.9	November-21	12.3	Undetermined
27	March-21	11.0	31.7	November-21	27.0	31.3
24	March-21	6.9	28.7	November-21	12.1	28.6
8	March-21	6.4	26.5	November-21	14.7	27.7
48	March-21	45.5	28.3	November-21	267.2	26.6
19	April-21	41.5	26.9	November-21	28.4	27.1
30	April-21	11.6	27.6	November-21	27.4	27.1
56	April-21	9.1	29.4	November-21	11.9	27.3
20	April-21	121.0	28.0	November-21	79.0	28.1
17	April-21	171.7	26.8	November-21	88.0	27.1
32	April-21	N/A	17.9	November-21	23602.0	17.1
61	April-21	50.8	26.9	November-21	123.6	24.5
57	April-21	35.7	29.1	November-21	15.8	26.8
84	April-21	7.3	35.8	November-21	3.3	Undetermined
50	April-21	33.8	29.6	November-21	47.2	27.7
58	July-21	41.2	31.4	November-21	9.1	29.1
9	July-21	260.4	23.8	November-21	502.0	25.0
28	July-21	197.5	23.0	November-21	870.3	22.5
73	July-21	333.3	24.9	November-21	N/A	21.5
5	July-21	3.9	34.4	November-21	below calibration range	36.6
75	July-21	70.8	22.4	November-21	N/A	18.5
16	July-21	9.9	29.0	November-21	21.0	29.3
13	July-21	791.7	19.8	November-21	N/A	20.2
54	July-21	41.4	28.4	November-21	N/A	26.3
21	August-21	2.8	29.8	November-21	N/A	29.8
2	August-21	3.9	27.5	November-21	N/A	32.9
44	August-21	1991.2	17.8	November-21	1156.0	16.3
25	August-21	103.2	25.4	November-21	56.2	25.2
68	August-21	110.2	26.7	November-21	372.2	23.8
51	August-21	2.5	27.2	November-21	19.3	25.3
10	August-21	N/A	12.7	November-21	41190.0	13.4
35	August-21	6.5	27.3	November-21	1.2	26.4
59	August-21	5.8	35.6	November-21	below calibration range	33.2
65	August-21	8.6	26.9	November-21	49.2	24.4
7	September-21	1478.8	20.5	November-21	N/A	21.8
15	September-21	N/A	16.6	November-21	N/A	16.9
34	September-21	65.7	24.9	November-21	N/A	24.0
43	September-21	62.5	25.2	November-21	N/A	23.8
39	September-21	28.2	25.5	November-21	N/A	24.3
38	September-21	57.5	26.2	November-21	N/A	25.1
40	September-21	1776.2	24.7	November-21	N/A	23.5
71	September-21	3.5	31.8	November-21	N/A	28.5

85	September-21	3.0	35.5	November-21	N/A	Undetermined
41	September-21	N/A	18.7	November-21	N/A	17.4
60	October-21	8.9	32.0	November-21	N/A	29.6
69	October-21	15.1	30.4	November-21	N/A	27.5
29	October-21	24.7	24.9	November-21	N/A	24.5
86	October-21	11.7	37.9	November-21	N/A	Undetermined
63	October-21	17.2	25.2	November-21	N/A	22.8
46	October-21	12.0	24.3	November-21	97.0	22.8
64	October-21	341.5	23.3	November-21	N/A	20.8
87	October-21	4.8	39.0	November-21	N/A	Undetermined
22	October-21	24.1	26.6	November-21	N/A	26.5
70	October-21	2318.7	21.8	November-21	N/A	18.7
