# Supplementary material for: SARS-CoV-2 reliably detected in frozen saliva samples stored up to one year
Source: PLoS One. 2022 Aug 11;17(8):e0272971. doi: 10.1371/journal.pone.0272971 (PMC9371282; doi:10.1371/journal.pone.0272971)
Supplement: S1 Table — (DOCX) [file pone.0272971.s002.docx]

| **Table S1.** Test and re-test Ct value differences by month from samples with complete data | | | | | | | | | |
| --- | --- | --- | --- | --- | --- | --- | --- | --- | --- |
| **Month** | **N** | **Mean Test N1 CT**  **(95% CI)** | **Mean Re-test N1 CT**  **(95% CI)** | **Absolute Difference**  **(95% CI)** | **Test Coefficient of Variation** | **Re-test Coefficient of Variation** | **Percent Change** | **Pearson Correlation (95% CI)** | **Intraclass Correlation (95% CI)** |
| Overall | 79 | 26.7  (25.7, 27.8) | 25.6  (24.5, 26.6) | -1.2  (-1.6, -0.7) | 17.1 | 17.9 | -4.1 | 0.89  (0.84, 0.93) | 0.87  (0.72, 0.93) |
| December ‘20 | 9 | 29.9  (25.9, 33.9) | 28.3  (24.0, 32.7) | -1.5  (-3.8, 0.8) | 17.5 | 20.1 | -5.4% | 0.85  (0.39, 0.96) | 0.83  (0.44, 0.96) |
| January  ‘21 | 8 | 28.2  (25.4, 30.9) | 26.3  (24.1, 28.5) | -1.8  (-4.5, 0.9) | 11.6 | 10.1 | -6.7% | 0.42  (-0.43, 0.86) | 0.37  (-0.25, 0.82) |
| February  ‘21 | 8 | 26.0  (22.1, 29.9) | 24.9  (21.2, 28.6) | -1.1  (-3.3, 1.1) | 17.8 | 17.8 | -4.2% | 0.83  (0.24, 0.96) | 0.82  (0.39, 0.96) |
| March  ‘21 | 9 | 28.0  (25.6, 30.4) | 27.1  (24.6, 29.6) | -0.9  (-2.0, 0.2) | 11.2 | 12.1 | -3.2% | 0.90  (0.56, 0.98) | 0.88  (0.53, 0.97) |
| April  ‘21 | 9 | 26.9  (21.2, 29.7) | 25.9  (23.2, 28.5) | -1.1  (-1.9, -0.2) | 13.2 | 13.3 | -3.7% | 0.95  (0.74, 0.99) | 0.91  (0.45, 0.98) |
| July  ‘21 | 9 | 26.4  (22.69, 30.0) | 25.5  (21.1, 29.8) | -0.9  (-2.5, 0.7) | 18.1 | 22.2 | -3.0% | 0.93  (0.67, 0.98) | 0.91  (0.68, 0.98) |
| August  ‘21 | 10 | 25.7  (21.2, 30.2) | 25.1  (20.5, 29.6) | -0.6  (-2.3, 1.1) | 24.5 | 25.5 | -2.3% | 0.93  (0.69, 0.98) | 0.93  (0.76, 0.98) |
| September ‘21 | 9 | 23.8  (20.3, 27.3) | 22.8  (20.0, 25.6) | -1.0  (-1.9, -0.02) | 19.1 | 16.1 | -4.2% | 0.98  (0.87, 0.99) | 0.93  (0.63, 0.99) |
| October  ‘21 | 8 | 26.1  (23.2, 29.0) | 24.1  (21.1, 27.2) | -1.9  (-2.9, -1.0) | 13.4 | 15.0 | -7.7% | 0.95  (0.72, 0.99) | 0.83  (-0.05, 0.97) |
