# Supplementary material for: SARS-CoV-2 reliably detected in frozen saliva samples stored up to one year
Source: PLoS One. 2022 Aug 11;17(8):e0272971. doi: 10.1371/journal.pone.0272971 (PMC9371282; doi:10.1371/journal.pone.0272971)
Supplement: S2 Table — (DOCX) [file pone.0272971.s003.docx]

| **Table S2.** Test and re-test antigen concentration differences by month excluding observations that were below the limit of detection | | | | | | | | | |
| --- | --- | --- | --- | --- | --- | --- | --- | --- | --- |
| **Month** | **N** | **Mean Test N1 CT**  **(95% CI)** | **Mean Re-test N1 CT**  **(95% CI)** | **Absolute Difference**  **(95% CI)** | **Test Coefficient of Variation** | **Re-test Coefficient of Variation** | **Percent Change** | **Pearson Correlation (95% CI)** | **Intraclass Correlation (95% CI)** |
| Overall | 51 | 352.6  (-45.5, 750.8) | 189.5  (93.9, 285.1) | -163.1  (-489.3, 163.1) | 401.5 | 179.3 | -46.3% | 0.81  (0.68, 0.88) | 0.37  (0.10, 0.58) |
| December ‘20 | 8 | 27.4  (-4.9, 59.7) | 59.1  (-15.9, 134.2) | 31.7  (-12.7, 76.2) | 141.2 | 151.8 | 115.7% | 0.97  (0.81, 0.99) | 0.66  (0.07, 0.92) |
| January  ‘21 | 7 | 1726.8  (-1648.7, 5102.4) | 555.3  (-28.0, 1138.6) | -1171.5  (-4022.6, 1679.6) | 211.4 | 113.6 | -67.8% | 0.92  (0.47, 0.99) | 0.31  (-0.47, 0.83) |
| February ‘21 | 8 | 274.2  (-35.3, 583.8) | 170.9  (54.1, 287.6) | -103.3  (-326.4, 119.7) | 135.0 | 81.7 | -37.7% | 0.83  (0.24, 0.96) | 0.54  (-0.14, 0.88) |
| March  ‘21 | 8 | 32.3  (-4.3, 68.8) | 45.0  (-30.3, 120.3) | 12.7  (-66.4, 91.9) | 135.7 | 200.1 | 39.3% | 0.13  (-0.64, 0.76) | 0.12  (-0.73, 0.74) |
| April  ‘21 | 9 | 53.6  (10.5, 96.8) | 47.2  (15.7, 78.7) | -6.4  (-39.4, 26.5) | 104.7 | 86.8 | -11.9% | 0.65  (-0.06, 0.91) | 0.64  (-0.01, 0.91) |
| July  ‘21 | 4 | 127.2  (-65.1, 319.6) | 350.6  (-310.8, 1012.0) | 223.4  (-290.3, 737.0) | 95.0 | 118.6 | 175.6% | 0.83  (-0.72, 0.995) | 0.39  (-0.43, 0.94) |
| August  ‘21 | 6 | 370.4  (-464.5, 1205.3) | 275.7  (-199.5, 750.8) | -94.7  (-492.0, 302.6) | 214.8 | 164.2 | -25.6% | 0.96  (0.65, 0.995) | 0.84  (0.27, 0.98) |
| October  ‘21 | 1 | 12.0 (--) | 97.0 (--) | 85 (--) | -- | -- | 708.3% | NA | NA |
| NOTE: Because there was 1 observation in October ’21, it was not possible to calculate certain values which is denoted by --. | | | | | | | | | |
