# Supplementary material for: SARS-CoV-2 reliably detected in frozen saliva samples stored up to one year
Source: PLoS One. 2022 Aug 11;17(8):e0272971. doi: 10.1371/journal.pone.0272971 (PMC9371282; doi:10.1371/journal.pone.0272971)
Supplement: Attachment — Submitted filename: Response to Reviewers 072822.docx [file pone.0272971.s005.docx]

July 28, 2022

*Principal Investigators*

Wilbur A. Lam, MD, PhD

Emory University

Georgia Institute of Technology

Children’s Healthcare of Atlanta

Greg Martin, MD, MSc

Emory University

Emory Healthcare

Grady Health System

Oliver Brand, PhD

Georgia Institute of Technology

Institute for Electronics and Nanotechnology

*Director of Operations*

Erika A. Tyburski, BS

Georgia Institute of Technology

*Part of the NIH*

*Point of Care Technology Research Network (POCTRN)*

*Supported by NIH-NIBIB*

Emily Chenette, PhD, Editor-in-Chief, PLOS ONE

Dear Dr. Chenette,

Thank you for suggesting a revision for our Original Investigation entitled, “SARS-CoV-2 reliably detected in frozen saliva samples stored up to one year,” we have responded to the requests below.

|  | Reviewer Comments | Author comments |
| --- | --- | --- |
| Editor comments | Full vendor details should include, company, city (state), and country | Completed |
|  | Tables should be in landscape not portrait layout | Completed |
|  | Conclusion should be in a separate section | Completed |
|  | Study strengths and limitations should be in a separate section, headed as addressed. It should be ahead of the conclusion.   We will only publish funding information present in the Funding Statement section of the online submission form. | Completed |
| Reviewer 4 | The authors have adequately responded to all of my comments except one - what went wrong with the January specimens? Understanding this seems very important to supporting the conclusions of the paper. Things look good for 8 of 9 months, but this is not a level that I generally would be willing to accept in the clinical laboratory, nor would I expect a regulatory agency to accept as adequate in any part of the developed world. An explanation, or at least a speculation on what went wrong with these samples seems very important to me. | We have addressed this in the new Strengths and Limitations section. |
| Reviewer 6 | Ln 90 what do you mean by simple random samples? I would suggest removing the word simple. | We have removed the word simple although simple random sample is a defined term and does have a meaning separate from just a “random sample”. The “simple” part means that all observations had equal chance of being selected. |

With best regards,

Joshua M Levy, MD, MPH, FACS

Associate Professor of Otolaryngology

Associate Vice Chair of Research

Emory University Department of Otolaryngology-HNS

Alternate Contact

Wilbur A. Lam

W. Paul Bowers Research Chair

Aflac Cancer & Blood Disorders Center at Children’s Healthcare of Atlanta

Emory University School of Medicine

Wallace H. Coulter Department of Biomedical Engineering

Emory University and Georgia Institute of Technology

The Atlanta Center for Microsystems-Engineered Point-of-Care Technologies
